# Therapeutic effect of postoperative adjuvant transcatheter arterial chemoembolization based on the neutrophil-to-lymphocyte ratio

**DOI:** 10.3389/fsurg.2022.1072451

**Published:** 2023-01-06

**Authors:** Guo-Ying Feng, Zheng-Rong Shi, Yu-Fei Zhao, Kai Chen, Jie Tao, Xu-Fu Wei, Yu Cheng

**Affiliations:** ^1^ Department of Hepatobiliary Surgery, The First Affiliated Hospital of Chongqing Medical University, Chongqing, China; ^2^ Department of Hepatobiliary Surgery, Daping Hospital, Army Medical University, Chongqing, China; ^3^ Nursing Department, University-Town Hospital of Chongqing Medical University, Chongqing, China

**Keywords:** hepatic carcinoma, neutrophil-to-lymphocyte ratio, postoperative adjuvant transcatheter arterial chemoembolization, prognosis, propensity score matching

## Abstract

**Aim:**

To evaluate the feasibility of the preoperative neutrophil-to-lymphocyte ratio (NLR) as an index to guide postoperative adjuvant transcatheter arterial chemoembolization (PA-TACE) in patients with liver cancer.

**Methods:**

We recruited a total of 166 patients with liver cancer who underwent surgery alone or surgery plus PA-TACE between January 2013 and June 2017 and compared the 1, 2, and 3-year recurrence-free survival (RFS) and overall survival (OS) between patients with high and low NLRs, surgery and surgery plus PA-TACE groups, and relevant subgroups using the Kaplan–Meier method. We also evaluated the independent factors affecting the prognosis of liver cancer after surgery using a Cox risk ratio model and correlation between NLR levels and high-risk recurrence factors of liver cancer with logistic regression analysis.

**Results:**

The 1, 2, and 3-year RFS rates were all significantly higher in the low-NLR group compared to the high-NLR group (*P* < 0.05). However, the 1, 2, and 3-year OS rates were similar in the low- and high-NLR groups (*P* > 0.05). After propensity score matching, the 1, 2, and 3-year RFS and OS rates were significantly better in patients treated with surgery plus PA-TACE compared with surgery alone (*P* < 0.05). The 1, 2, and 3-year RFS and OS rates were also significantly better in the surgery plus PA-TACE subgroup compared with the surgery-alone subgroup in the high-NLR group (*P* < 0.05), but there was no significant difference in RFS or OS between the surgery plus PA-TACE and surgery-alone subgroups at 1, 2, and 3 years in the low-NLR group (*P* > 0.05). Multivariate analysis in the high-NLR group showed that a poorly differentiated or undifferentiated tumor was an independent risk factor for postoperative RFS. Multiple tumors were an independent risk factor for postoperative OS (*P* < 0.05), while PA-TACE was an independent protective factor for postoperative RFS and OS (*P* < 0.05). In the low-NLR group, AFP > 400 µg/L was an independent risk factor for postoperative OS (*P* < 0.05). Multivariate logistic regression indicated that patients with a maximum tumor diameter of >5 cm were at increased risk of having high NLR levels compared to patients with a maximum tumor diameter of <5 cm (*P* < 0.05).

**Conclusion:**

PA-TACE can improve the prognosis of patients with a high preoperative NLR (≥2.5), but has no obvious benefit in patients with low preoperative NLR (<2.5). This may provide a reference for clinical selection of PA-TACE.

## Introduction

Radical hepatectomy is currently one of the most effective treatments for liver cancer; however, the 5-year overall survival (OS) rate is still not ideal, ranging from 25%–55%, largely due to the high postoperative recurrence rate (60%–100%) (1). TNM staging, positive margins, and microvascular infiltration are among the factors known to affect recurrence after radical hepatectomy (2). Previous studies showed that the median survival of patients with liver cancer recurrence was 54 months less than that of patients without recurrence (3), indicating the need for postoperative adjuvant treatment of liver cancer in patients with high-risk recurrence factors. Transcatheter arterial chemoembolization (TACE) is one of the main treatments for patients with advanced hepatocellular carcinoma (HCC), and is also widely recommended for the preoperative and postoperative treatment of patients undergoing radical hepatectomy (4). Several studies (5–7) have shown that postoperative adjuvant TACE (PA-TACE) can improve recurrence-free survival (RFS) and OS, and can significantly improve the prognosis of HCC patients. However, the indicators used to identify the postoperative liver cancer patients likely to benefit from PA-TACE are limited, and the indications for PA-TACE differ among medical centers. Further studies are therefore needed to identify potential indicators to evaluate the effect of PA-TACE, and to develop comprehensive and accurate standards to guide clinical decision-making, reduce tumor recurrence in patients after HCC surgery, and improve survival through individualized treatment.

The neutrophil-to-lymphocyte ratio (NLR), as an indicator of systemic inflammation, has been shown to be related to the prognosis of HCC patients, with a higher NLR predicting a worse prognosis (5, 8, 9). The mechanism may be related to the complex nature of the tumor itself and the interaction between the tumor and tumor microenvironment, coupled with the role of the chronic inflammatory environment in the occurrence and development of liver cancer (10). TACE-induced ischemia and hypoxia affect the activity of immune cells, leading to changes in the inflammatory environment. Although previous studies showed that a high NLR was related to a poor prognosis in PA-TACE-treated liver cancer patients (8), the causative role of PA-TACE in this poor prognosis is unclear.

In this study, we collected clinical data for patients with liver cancer treated with radical hepatectomy alone or with radical hepatectomy plus PA-TACE to investigate the correlation between their prognosis and preoperative NLR level. We further explored the possibility of using NLR as an index to screen for patients suitable for postoperative PA-TACE in order to provide a reference for choosing TACE after radical hepatectomy. We also analyzed the independent factors affecting the prognosis of liver cancer after surgery and performed a preliminary study on the correlation between NLR levels and high-risk recurrence factors of liver cancer and between PA-TACE frequency and RFS in patients with liver cancer.

## Materials and methods

### Study patients

We retrospectively analyzed the clinical data for patients with liver cancer who underwent surgery at the Liver Center of the First Affiliated Hospital of Chongqing Medical University, Chongqing, China between January 2013 and June 2017. The inclusion criteria (11–13) were as follows: (1) preoperative liver function Child–Pugh score A/B, and liver reserve function indicating sufficient residual liver volume; (2) not accompanied by tumor rupture or any signs of sepsis; (3) radical liver tumor resection, and postoperative medical examination confirming no cancer cells in the resection margin; (4) HCC confirmed by postoperative medical examination and immunohistochemical analysis; (5) no portal vein or other large blood vessel invasion or distant metastasis; and (6) patients in the intervention group who received one or two TACE treatments after surgery. The exclusion criteria (11, 13, 14) were as follows: (1) tumor recurrence demonstrated within 2 months after surgery or during TACE therapy; (2) complicated with other tumors; (3) adjuvant treatments other than TACE performed during the interval between the first diagnosis of recurrence or metastasis after surgery; (4) loss to follow-up in <1 year; and (5) diseases with abnormal blood cell counts caused by blood, immune system, or other non-liver cancer causes. We collected clinical data [age, sex, history of hepatitis B, cirrhosis, preoperative blood routine, liver function, alfa-fetoprotein (AFP) levels] and tumor data [tumor size, number of lesions, degree of differentiation, microvascular invasion (MVI) grade]. The tumor data were issued by the Imaging Center of the First Affiliated Hospital of Chongqing Medical University and the Clinicopathology Department of the Molecular Medicine Testing Center of Chongqing Medical University.

### Ethics and informed consent

This retrospective study was conducted in line with the ethical guidelines of the Declaration of Helsinki revised in 1975, and was approved by the Ethics Committee of the First Affiliated Hospital of Chongqing Medical University (No. 2019-021).

### Therapies

Patients who met the conditions for surgery underwent segmental hepatectomy, hemihepatectomy, or tumor resection according to the size, location, and distribution of the tumor. 1–1.5 months after surgery, patients at high risk of recurrence (e.g., maximum lesion diameter >5 cm, MVI > M1, and less-differentiated tumor) received TACE after multidisciplinary discussion and evaluation. Seldinger technology was used to puncture the catheter through the femoral artery under local anesthesia with 1% lidocaine. PA-TACE was conducted for the entire remnant liver of these postoperative patients under the guidance of hepatic and CT angiography. Firstly, tiny doses (0.5–1.0 ml) of lipiodol were used to observe if deposition occur in the remnant liver. For patients with lipiodol deposition, chemotherapeutic agents (oxaliplatin, irinotecan, fluorouracil, epirubicin, or pirarubicin) and lipiodol were used to chemoembolize the corresponding artery after assessing the drug dose based on body surface area and liver function. TACE without lipiodol would be performed for these without lipiodol deposition. After treatment, radiography was conducted again to evaluate the effect of embolization if necessary. A total of one or two TACE treatments were given with an interval of ≥3 weeks, and liver function was assessed before surgery to confirm the patient's ability to withstand interventional therapy. None of the patients with HCC received any other types of adjuvant therapy, such as targeted therapy, immunotherapy, or absolute alcohol injection, from the time of surgery to the first diagnosis of recurrence or metastasis.

### Follow-up

All patients were followed-up regularly in the outpatient clinic (every 3 months in the first year after surgery and every 6 months thereafter). Outpatient follow-up evaluations included liver and kidney function tests, hepatitis B virus-DNA quantification, tumor markers, abdominal color Doppler ultrasound, or enhanced computed tomography of the abdomen and chest. The follow-up endpoint was June 20, 2020 and the median follow-up time was 47 months [95% confidence interval (CI): 43.2–50.7 months]. The diagnostic criteria for tumor recurrence were consistent with the initial diagnostic criteria for HCC. The follow-up endpoint of the study was the patient's time of death or loss to follow-up.

### Statistical analysis

Statistical analysis was carried out using SPSS 22.0 (IBM Corp., Armonk, NY, USA). The study endpoint was OS rate, based on death from any cause from the time of first surgery. Propensity score matching (PSM) was used to reduce the differences in baseline data between the groups. The groups were matched according to the 1:1 nearest-matching method, with the caliper value set to 0.3. Results were compared between groups using *χ*^2^ and number-rank tests, survival analyses were carried out using the Kaplan–Meier method, independent factors affecting prognosis were analyzed using a Cox proportional risk model, and the correlation between high-risk factors for recurrence and NLR levels was analyzed by logistic regression. *P* < 0.05 was considered statistically significant.

## Results

### Baseline patient information

A total of 166 patients were enrolled in this study, of whom 85 underwent radical hepatectomy alone and 81 underwent radical hepatectomy combined with PA-TACE. There were no significant differences between the two groups in terms of sex, hepatitis B history, preoperative liver function, preoperative AFP levels, and preoperative NLR levels. The surgery-alone group included higher proportions of patients aged >55 years, with liver cirrhosis, and with single tumors. After PSM to eliminate differences, 80 pairs of patients were successfully matched with no significant differences in any baseline items between the groups (*P* > 0.05) (Table 1). Based on a previous study (15) and the median NLR in the current study, we divided the enrolled cases into a high-NLR group (NLR ≥ 2.5) and low-NLR group (NLR < 2.5). There were no significant differences in any items between the groups (*P* > 0.05) (Table 2). The surgery-alone and surgery plus PA-TACE groups were also further divided into high-NLR and low-NLR subgroups. There were 45 patients in the high-NLR and 40 in the low-NLR subgroup in the surgery-alone group, and 39 and 42, respectively, in the surgery plus PA-TACE subgroup, with no significant differences in any items between the paired subgroups (Tables 3, 4). The high-NLR and low-NLR groups were also divided into surgery and surgery plus PA-TACE subgroups. The high-NLR group included 45 patients in the surgery-alone subgroup and 39 in the surgery plus PA-TACE subgroup, and the low-NLR group included 40 and 42 in the surgery-alone and surgery plus PA-TACE subgroups, respectively, with no significant differences in any items between the subgroups (Tables 5, 6).

**Table 1:** Basic characteristics of patients in terms of treatment options [cases (%)].

Characteristic	Pre-PSM			After PSM		
	Operating group (*n* = 85)	Operation + TACE group (*n* = 81)	*P*	Operating group (*n* = 80)	Operation + TACE group (*n* = 80)	*P*
Age (>55)	49 (57.6)	32 (39.5)	0.019	44 (55.0)	32 (40.0)	0.057
Gender (male)	75 (88.2)	70 (86.4)	0.725	70 (87.5)	69 (86.3)	0.815
Hepatitis B history	72 (84.7)	73 (90.1)	0.294	70 (87.5)	72 (90.0)	0.617
Liver cirrhosis	61 (71.8)	45 (55.6)	0.030	56 (70.0)	44 (55.0)	0.050044
Pre-operative liver function (A)	84 (98.8)	79 (97.5)	0.966	79 (98.8)	78 (97.5)	1.000
AFP (>400 µg/L)	21 (24.7)	21 (25.9)	0.857	21 (26.25)	21 (26.25)	1.000
Number of lesions (single)	76 (89.4)	63 (77.8)	0.042	71 (88.8)	63 (78.8)	0.086
Maximum diameter of lesions (>5 cm)	47 (55.3)	42 (51.9)	0.657	42 (52.5)	41 (51.3)	0.874
NLR (≥2.5)	45 (53.0)	39 (48.1)	0.537	40 (50.0)	38 (47.5)	0.752

AFP, alpha foetoprotein; NLR, neutrophil-to-lymphocyte ratio; PSM, propensity score matching; TACE, transcatheter arterial chemoembolization.

**Table 2:** Basic characteristics of patients in terms of NLR level [cases (%)].

Characteristic	high NLR (*n* = 84)	low NLR (*n* = 82)	*P*
Age (>55)	38 (45.2)	43 (52.4)	0.353
Gender (male)	76 (90.5)	69 (84.1)	0.220
Hepatitis B history	74 (88.1)	71 (86.6)	0.770
Liver cirrhosis	53 (63.1)	53 (64.6)	0.837
Pre-operative liver function (A)	83 (98.8)	80 (97.6)	0.983
AFP (>400 µg/L)	22 (26.2)	20 (24.4)	0.790
Number of lesions (single)	69 (82.1)	70 (85.4)	0.574
Treatment programs (Operation + TACE)	39 (46.4)	42 (51.2)	0.537

AFP, Alpha foetoprotein; NLR, Neutrophil-to-lymphocyte ratio.

**Table 3:** Basic characteristics of surgery-alone subgroup.

Characteristic	high NLR (*n* = 45)	low NLR (*n* = 40)	*P*
Age (>55)	25	24	0.679
Gender (male)	42	33	0.226
Hepatitis B history	37	35	0.500
Liver cirrhosis	29	32	0.112
Pre-operative liver function (A)	45	39	0.471
AFP (>400 µg/L)	9	12	0.286
Number of lesions (single)	39	37	0.604

AFP, Alpha foetoprotein; NLR, Neutrophil-to-lymphocyte ratio.

**Table 4:** Basic characteristics of surgery plus PA-TACE subgroup.

Characteristic	high NLR (*n* = 39)	low NLR (*n* = 42)	*P*
Age (>55)	13	19	0.273
Gender (male)	34	36	0.847
Hepatitis B history	37	36	0.314
Liver cirrhosis	24	21	0.296
Pre-operative liver function (A)	38	41	1.000
AFP (>400 µg/L)	13	8	0.143
Number of lesions (single)	30	33	0.858

AFP, Alpha foetoprotein; NLR, Neutrophil-to-lymphocyte ratio.

**Table 5:** Basic characteristics of high-NLR subgroup.

Characteristic	Operating group (*n* = 45)	Operation + TACE group (*n* = 39)	*P*
Gender (male)	42	34	0.558
Hepatitis B history	37	37	0.148
Pre-operative liver function (A)	45	38	0.464
AFP (>400 µg/L)	9	13	0.166
Number of lesions (single)	39	30	0.245

AFP, Alpha foetoprotein; TACE, Transcatheter arterial chemoembolization.

**Table 6:** Basic characteristics of low-NLR subgroup.

Characteristic	Operating group (*n* = 40)	Operation + TACE group (*n* = 42)	*P*
Gender (male)	33	36	0.690
Hepatitis B history	35	36	0.813
Pre-operative liver function (A)	39	41	1.000
AFP (>400 µg/L)	12	8	0.248
Number of lesions (single)	37	33	0.074

AFP, Alpha foetoprotein; TACE, Transcatheter arterial chemoembolization.

In the surgery plus PA-TACE group, subgroups were created using PA-TACE frequency. The proportion of patients with AFP > 400 µg/L in the one-time group (*n* = 50) was significantly higher than that in the two-time group (*n* = 31; *P* < 0.05). After PSM to eliminate the differences, 31 pairs of patients were successfully matched with no significant differences in any baseline items between the groups (*P* > 0.05; Table 7). Patients were then divided by the frequency of PA-TACE in the high- and low-NLR subgroups of the surgery plus PA-TACE group. In the low-NLR subgroup, the proportion of patients with MVI > M1 in the one-time group (*n* = 22) was significantly lower than that in the two-time group (*n* = 20; *P* < 0.05). After PSM, 19 pairs of patients were successfully matched with no significant differences in any baseline items between the groups (*P* > 0.05). In the high-NLR subgroup, there was no significant difference in each item between the one-time group (*n* = 28) and two-time group (*n* = 11; *P* > 0.05; Table 8).

**Table 7:** Basic characteristics of patients in terms of PA-TACE frequency in surgery plus PA-TACE group.

Characteristic	Operation + TACE group			After PSM		
	One-time (*n* = 50)	Two-time (*n* = 31)	*P*	One-time (*n* = 31)	Two-time (*n* = 31)	*P*
Age (>55)	20	12	0.908	13	12	0.796
Gender (male)	45	25	0.389	28	25	0.471
Hepatitis B history	47	26	0.270	29	26	0.422
Liver cirrhosis	27	18	0.720	15	18	0.445
Pre-operative liver function (A)	49	30	1.000	30	30	1.000
AFP (>400 µg/L)	17	4	0.035	8	4	0.199
Number of lesions (single)	41	22	0.246	26	22	0.224
MVI (>M1)	34	27	0.053	23	27	0.409

AFP, Alpha foetoprotein; MVI, microvascular invasion; PSM, Propensity score matching; TACE, Transcatheter arterial chemoembolization.

**Table 8:** Basic characteristics of patients in terms of PA-TACE frequency in high- and low-NLR subgroup of surgery plus PA-TACE group.

Characteristic	Low-NLR subgroup			High-NLR subgroup			Low-NLR subgroup (after PSM)		
	One-time (*n* = 22)	Two-time (*n* = 20)	*P*	One-time (*n* = 28)	Two-time (*n* = 11)	*P*	One-time (*n* = 19)	Two-time (*n* = 19)	*P*
Age (>55)	8	11	0.226	12	1	0.102	6	10	0.189
Gender (male)	20	16	0.570	25	9	0.924	17	15	0.656
Hepatitis B history	20	16	0.570	27	10	0.490	17	16	1.000
Liver cirrhosis	9	12	0.217	18	6	0.844	8	11	0.330
Pre-operative liver function (A)	21	20	1.000	28	10	0.282	18	19	1.000
AFP (>400 µg/L)	7	1	0.069	10	3	0.900	6	1	0.094
Number of lesions (single)	20	13	0.095	21	9	0.974	17	12	0.127
MVI (>M1)	12	17	0.033	22	10	0.660	12	16	0.141

AFP, Alpha foetoprotein; MVI, microvascular invasion; NLR, Neutrophil-to-lymphocyte ratio; PSM, Propensity score matching.

### Results of survival analysis in every group and subgroup

#### Effect of the NLR level on RFS and OS in patients after HCC treatment

**Figure 1:**
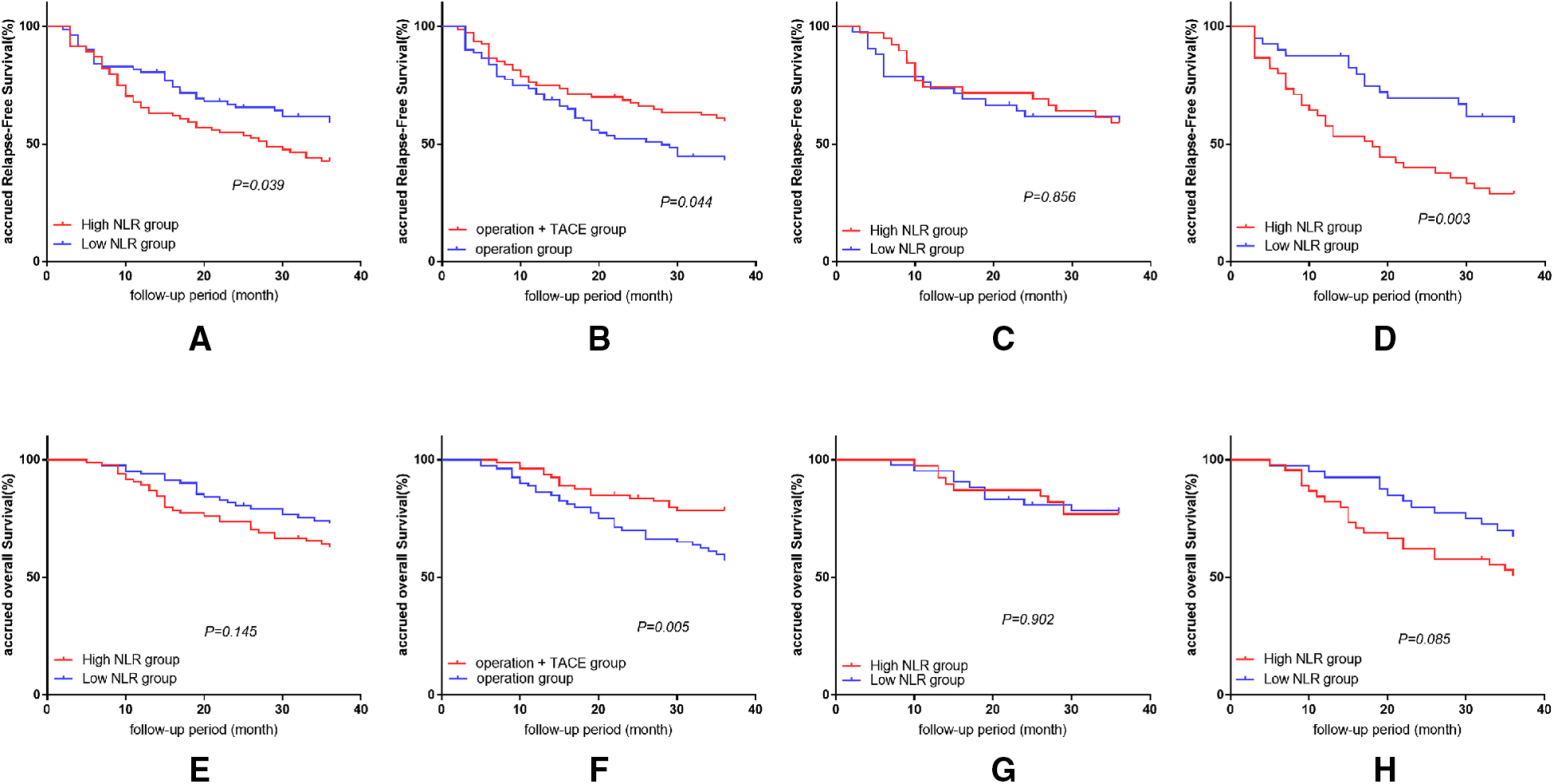
Results of survival analysis in every group and subgroup. (**A**) Effect of NLR level on RFS in patients after HCC treatment. (**B**) Effect of treatment methods on RFS after liver cancer surgery. (**C**) Comparison of RFS in patients with high and low NLR in surgery plus PA-TACE group. (**D**) Comparison of RFS in patients with high and low NLR in surgery-alone group. (**E**) Effect of NLR level on OS in patients after HCC treatment. (**F**) Effect of treatment methods on OS after liver cancer surgery. (**G**) Comparison of OS in patients with high and low NLR in surgery plus PA-TACE group. (**H**) Comparison of OS in patients with high and low NLR in surgery-alone group.

The 1, 2, and 3-year RFS rates were significantly higher in the low-NLR compared with the high-NLR group (80.5%, 65.6%, and 59.1% vs. 65.5% 54.8%, and 42.9%, respectively; *P* = 0.039). The 1, 2, and 3-year OS rates were also higher in the low-NLR than the high-NLR group, but the differences were not significant (93.9%, 80.5%, and 72.9% vs. 89.3%, 73.8%, and 63.0%, respectively; *P* = 0.145; Figures 1A,E).

#### Effect of treatment methods on RFS and OS after liver cancer surgery

After eliminating the differences between the groups by PSM, the 1, 2, and 3-year RFS and OS rates were significantly better in the surgery plus PA-TACE compared with the surgery-alone group (RFS: 75.0%, 67.5%, and 59.7% vs. 71.3%, 52.2%, and 43.2%, respectively; *P* = 0.044; OS: 96.3%, 83.7%, and 78.6% vs. 86.3%, 70.0%, and 57.2%, respectively; *P* = 0.005; Figures 1B,F).

#### Comparison of RFS and OS in patients with high and low NLR under the same treatment

In the surgery plus PA-TACE group, there was no significant difference in RFS or OS at 1, 2, and 3 years between the high-NLR and low-NLR subgroups (RFS: 74.4%, 71.8%, and 59.0% vs. 73.8%, 61.7%, and 59.2%, respectively; *P* = 0.856; OS: 97.4%, 87.2%, and 76.9% vs. 95.2%, 80.9%, and 78.4%, respectively; *P* = 0.902; Figures 1C,G).

In the surgery-alone group, the 1, 2, and 3-year RFS rates were significantly higher in the low-NLR subgroup compared with the high-NLR subgroup (87.5%, 69.5%, and 59.1% vs. 57.8%, 40.0%, and 28.9%, respectively; *P* = 0.003). The 1, 2, and 3-year OS rates were also higher in the low-NLR than in the high-NLR subgroup, but the difference was not significant (92.5%, 80.0%, and 67.3 vs. 82.2%, 62.2%, and 50.8%, respectively; *P* = 0.085; Figures 1D,H).

### Comparison of RFS and OS in treatment subgroups with the same level of NLR

**Figure 2:**
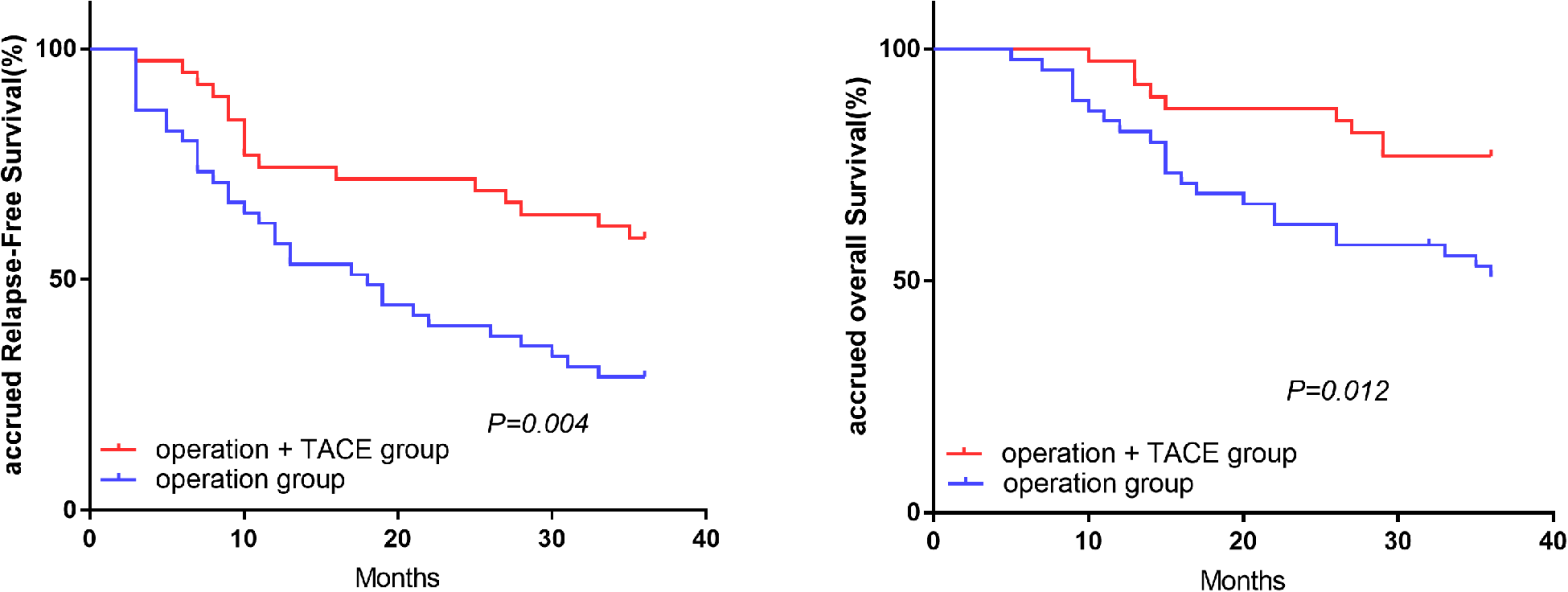
Effect of treatment methods on RFS and OS in high-NLR group.

**Figure 3:**
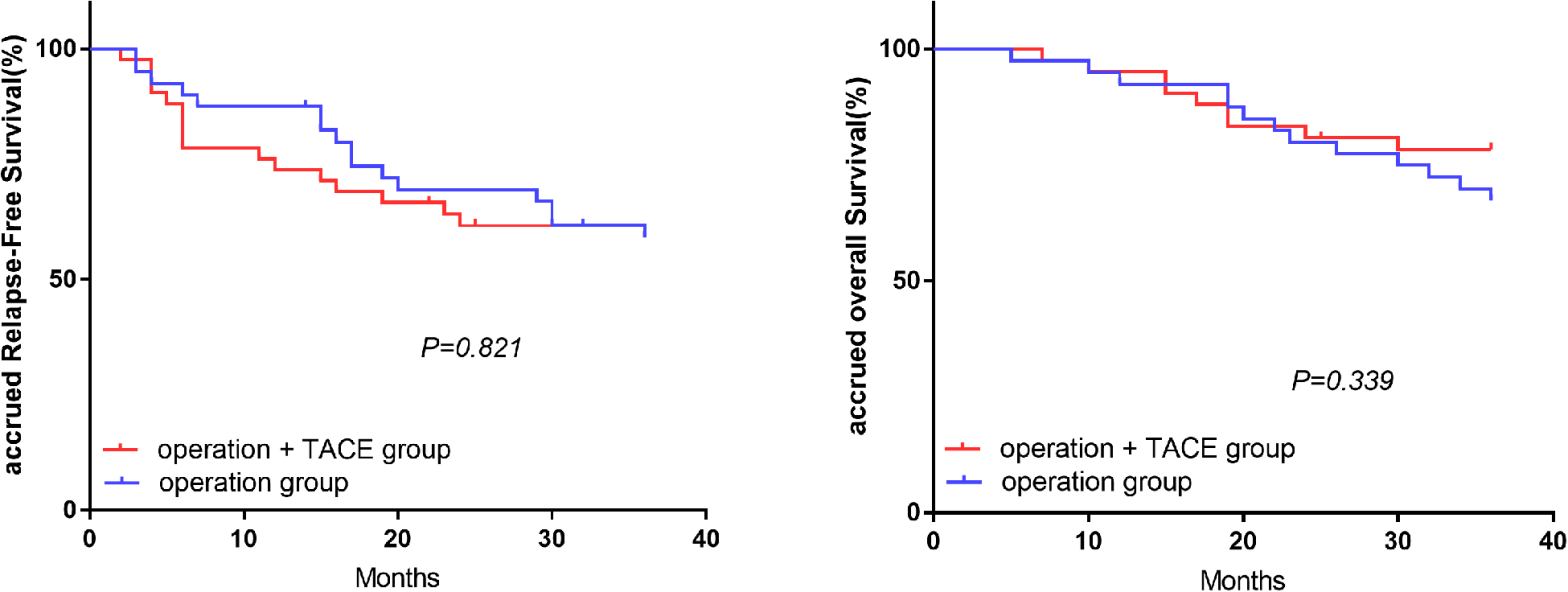
Effect of treatment methods on RFS and OS in low-NLR group.

In the high-NLR group, the 1, 2, and 3-year RFS and OS rates were significantly higher in the surgery plus PA-TACE compared with the surgery-alone subgroup (RFS: 74.4%, 71.8%, and 59.0% vs. 57.8%, 40.0%, and 28.9%, respectively; *P* = 0.004; OS: 97.4%, 87.2%, and 76.9% vs. 82.2%, 62.2%, and 50.8%, respectively; *P* = 0.012; Figure 2). However, in the low-NLR group, there was no significant difference in RFS or OS between the surgery plus PA-TACE subgroup and surgery-alone subgroups at 1, 2, and 3 years (RFS: 73.8%, 61.7%, and 59.2% vs. 87.5%, 69.5%, and 59.1%, respectively; *P* = 0.821; OS: 95.2%, 80.9%, and 78.4% vs. 92.5%, 80.0%, and 67.3%, respectively; *P* = 0.339; Figure 3).

### Multivariate analysis of recurrence and survival in patients with HCC after surgery

After PSM matching in the high-NLR group, multivariate analysis showed that a poorly differentiated or undifferentiated tumor was an independent risk factor for postoperative RFS, and multiple tumors was an independent risk factor for postoperative OS [hazard ratio (HR) = 2.10, *P* = 0.011 and HR = 3.73, *P* = 0.001, respectively], while PA-TACE was an independent protective factor for postoperative RFS and OS (HR = 0.46 and 0.32, *P* = 0.012 and 0.004, respectively). In the low-NLR group, AFP > 400 µg/L was an independent risk factor for postoperative RFS and OS (HR = 2.02 and 3.70, *P* = 0.044 and 0.003, respectively; Table 9).

**Table 9:** Multivariate analysis of recurrence and survival in patients with HCC after surgery.

Factors	High-NLR group				Low-NLR group			
	RFS		OS		RFS		OS	
	HR (95%CI)	*P*	HR (95%CI)	*P*	HR (95%CI)	*P*	HR (95%CI)	*P*
Hepatitis B history	–	–	–	–	–	–	–	–
Liver cirrhosis	–	–	–	–	–	–	–	–
AFP (>400 µg/L)	–	–	–	–	–	–	3.23 (1.39–7.50)	0.006
Number of lesions (multiple)	–	–	3.73 (1.76–7.92)	0.001	–	–	–	–
Maximum diameter of lesions (>5 cm)	–	–	–	–	–	–	–	–
Method	0.46 (0.25–0.84)	0.012	0.32 (0.14–0.70)	0.004	–	–	–	–
Differential	2.10 (1.18–3.74)	0.011	–	–	–	–	–	–

AFP, Alpha foetoprotein; NLR, Neutrophil-to-lymphocyte ratio.

### Logistic regression analysis of correlation between high-risk factors for recurrence of liver cancer and NLR

Among several high-risk recurrence factors, such as AFP > 400 µg/L, multiple tumors, maximum tumor diameter >5 cm, and poorly differentiated tumors, multivariate logistic regression identified maximum tumor diameter >5 cm as being correlated with the NLR level. Patients with a maximum tumor diameter >5 cm were at increased risk of having high NLR levels compared with patients with a maximum tumor diameter <5 cm (OR = 5.12, 95% CI: 2.64–9.92, *P* < 0.05).

### Effect of PA-TACE frequency on RFS in patients after HCC treatment

**Figure 4:**
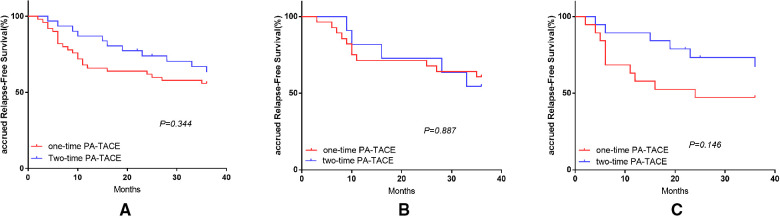
Effect of PA-TACE frequency on RFS in HCC patients in surgery plus PA-TACE group and its subgroups. (**A**) Comparison of RFS in patients with one-time and two-time PA-TACE in surgery plus PA-TACE group. (**B**) Comparison of RFS in patients with one-time and two-time PA-TACE in the high NLR subgroup. (**C**) Comparison of RFS in patients with one-time and two-time PA-TACE in the low NLR subgroup.

**Figure 5:**
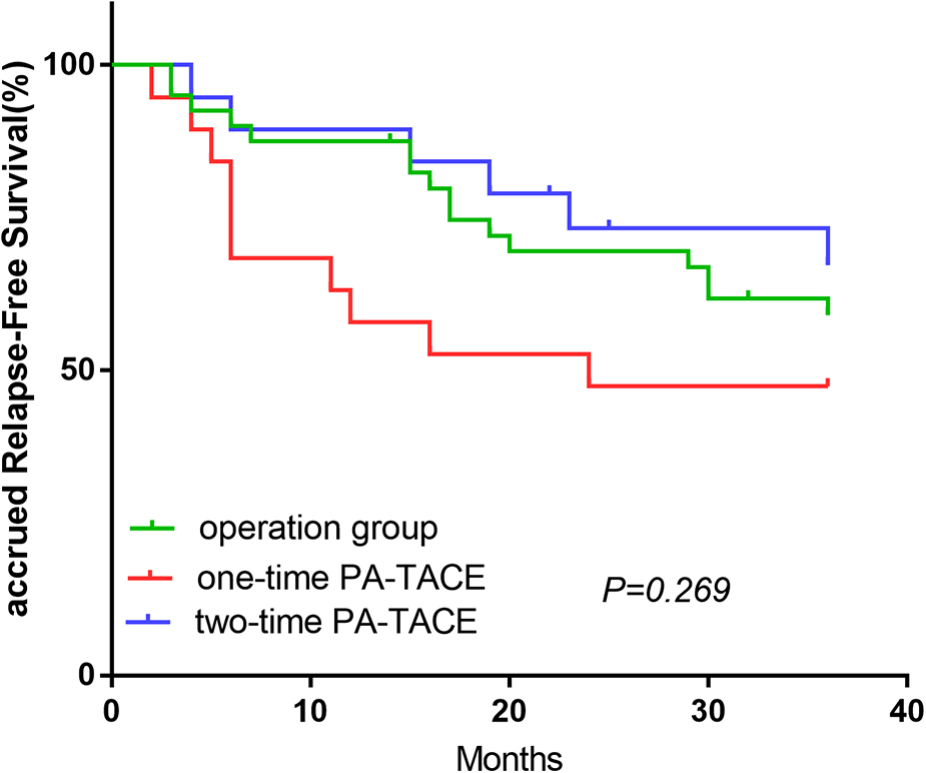
Comparison of operation and one- and two-time PA-TACE groups in low-NLR group.

The OS-related survival analysis was not performed due to the small number of cases in each group and the disparity in the number of cases with OS endpoints between groups. Only survival analysis for RFS at 1, 2, and 3 years was performed. In the surgery plus PA-TACE group, there was no statistically significant difference in RFS at 1, 2, and 3 years between one- and two-time PA-TACE groups (66.0%, 62.0%, and 56% vs. 87.1%, 74.1%, and 63.5%, respectively; *P* = 0.344; Figure 4A). In the high- and low-NLR subgroups, there was no statistically significant difference in RFS at 1, 2, and 3 years between one- and two-time PA-TACE groups (High-NLR group: 71.4%, 67.9%, and 60.7% vs. 81.8%, 72.7%, and 54.5%, respectively; *P* = 0.887; Low-NLR group: 57.9%, 47.4%, and 47.4% vs. 89.5%, 73.3%, and 67.2%, respectively; *P* = 0.146; Figures 4B,C). In the low-NLR group, no statistically significant difference was found in 1, 2, and 3-year RFS among the surgery, one-time PA-TACE, and two-time PA-TACE groups (87.5%, 69.5%, and 59.1% vs. 57.9%, 47.4%, and 47.4% vs. 89.5%, 73.3%, and 67.2%, respectively; *P* = 0.269; Figure 5).

## Discussion

The high recurrence of tumors after radical hepatectomy means that postoperative adjuvant therapy plays a significant role in improving the patient prognosis. Yang et al. (16) showed that postoperative adjuvant treatments such as PA-TACE, radiotherapy, and sorafenib improved the prognosis of patients with microvascular invasion after surgery. Clinical research into the indications of PA-TACE after liver cancer surgery is currently focused mainly on postoperative tumor-related indicators such as MVI grade, tumor volume, tumor number, tumor differentiation, and Ki-67 (11, 17, 18). A few laboratory indicators, such as AFP, serum gamma-glutamyl transferase, and the ferritin/hemoglobin ratio, have also been related to prognosis (5, 18), but are not widely used. This has led clinicians to mainly use PA-TACE for patients with a high risk of recurrence (large tumor diameter, multiple tumors, MVI positive, and poor tumor differentiation) according to postoperative tumor-related indicators. The proportion of patients with a low risk of recurrence receiving PA-TACE is small, and the benefit of PA-TACE in these patients is unclear. Xie et al. (19) found that PA-TACE may benefit patients with a low risk of recurrence more than patients with a high risk of recurrence. However, a meta-analysis by Chen et al. (18) showed that PA-TACE did not improve the prognosis among patients with a tumor diameter ≤5 cm, single tumor, or MVI negative, and may even have promoted postoperative recurrence in some patients. We therefore aimed to address this controversy by examining the value of NLR for predicting the prognosis of patients with liver cancer treated with surgery combined with PA-TACE.

The relationship between NLR and cancer progression has been demonstrated in numerous studies (9, 20, 21), with a high NLR related to the recurrence and poor prognosis of HCC. The upregulation of inflammatory pathways in the body under a high NLR environment leads to more aggressive tumor behaviors (9, 20). One reason for the increase in NLR is an increase in neutrophils, which leads to an increased production of neutrophil-derived cytokines, such as vascular endothelial growth factor (VEGF), matrix metalloproteinases, and interleukin-18. VEGF promotes angiogenesis and matrix metalloproteinases exacerbate inflammation and exudation, while interleukin-18 damages the function of NK cells and T cells, thereby impairing the host's immune response to tumor antigens (22–24). A decrease in lymphocytes also increases the NLR and is another important factor in suppressing cell-mediated immunity (25), which mainly depends on lymphocytes. A large number of lymphocytes in the tumor site has been associated with a good prognosis, and a decrease in lymphocytes may lead to tumor cell immune escape, which is a predictor of poor prognosis (26, 27). At the same time, neutrophils can inhibit lymphocyte-mediated cell lysis, further aggravating the poor prognosis (28).

We conducted this retrospective study in 166 patients, and the 1, 2, and 3-year RFS and OS rates were all significantly better in the surgery plus PA-TACE group compared with the surgery-alone group. This was similar to the results of previous studies (29–31), and thus confirmed the effect of PA-TACE in patients with liver cancer. The 1, 2, and 3-year RFS rates were significantly better in the low-NLR compared with the high-NLR group. Although the 1, 2, and 3-year OS rates showed no significant difference between the low- and high-NLR groups, the survival curves suggested that the low-NLR group still had a better prognosis. Multivariate analysis of the subgroups showed that PA-TACE was an independent protective factor for postoperative RFS and OS among patients with a high NLR, but this effect did not apply to patients with a low NLR.

These differences between the subgroups were maintained after further stratification by the NLR level: the prognosis in the surgery plus PA-TACE group was significantly superior to that in the surgery-alone group in the high-NLR group, while the prognoses of the two subgroups were similar in the low-NLR group. After stratification by treatment, a low NLR was associated with a better prognosis than a high NLR in the surgery-alone group, and the difference in OS between the two subgroups was more obvious than the difference in OS between the high- and low-NLR groups. The prognoses of the patients with high and low NLRs treated with surgery plus PA-TACE were similar. Combined with the results of multivariate analysis, we speculated that the addition on PA-TACE had no significant effect on the prognosis of patients in the low-NLR group. Whether the prognosis deteriorated in the surgery plus PA-TACE subgroup or improved in the surgery-alone subgroup, the results indicated that PA-TACE cannot benefit patients with a relatively low NLR. This inference can also explain the lack of difference in prognosis between patients with high and low NLRs in the surgery plus PA-TACE group, if PA-TACE worsens the prognosis of patients in the low NLR subgroup. This difference may be due not only to a unilateral change in the low NLR subgroup, but also an improvement in the prognosis of the high NLR subgroup treated by surgery combined with PA-TACE.

In the study of PA-TACE frequency, we found no statistically significant difference in RFS at 1, 2, and 3 years between the two groups, which might indicate that for patients with HCC, undergoing PA-TACE one or two times has a similar impact on their prognosis. This effect was particularly pronounced in the high-NLR group, suggesting that for high-NLR patients with a high risk of recurrence, two-time PA-TACE may not lead to a better outcome. However, in the low-NLR group, although the 1, 2, and 3-year RFS of one- and two-time PA-TACE groups was also not statistically different, the two-time PA-TACE group showed a significant advantage, which was no longer present when compared to the surgery group.

We speculate that the reason for this phenomenon in the low-NLR environment is that the ratio of neutrophils to lymphocytes was lower under low NLR conditions, indicating that the anti-tumor effect (mediated by antitumor-associated lymphocytes, including CD8 + T (32, 33) and natural killer T cells (34–36)) in the tumor microenvironment is to a certain extent better than the tumor-promoting effect [mediated by the monocyte-macrophage system (37, 38)]. Multiple TACE procedures kill tumor cells and at the same time help to activate the anti-tumor immune response, so that the body maintains an imbalanced state in which the anti-cancer effect is superior to promoting cancer-promoting effect for a long time, which is beneficial to obtain a better RFS. However, due to the relative lack of quantity and quality in our patient data under this grouping, this result still needs to be further confirmed by prospective studies with large samples.

One of the main reasons for the recurrence of liver cancer within 2 years after surgery is the presence of invisible intrahepatic metastases before surgery, resulting in many radical operations failing to achieve a radical cure (17, 39). PA-TACE can combat the possible surviving tumor cells through high postoperative local perfusion of chemotherapeutic drugs and selective vascular embolization to prevent possible micrometastases and recurrence (19). However, the hypoxic environment after interventional embolization can also stimulate the expression of hypoxia-inducible factor-1α and VEGF, activate related signal pathways, induce angiogenesis, and form a microenvironment that is conducive to tumor growth, thus promoting tumor recurrence, growth, and metastasis (40). PA-TACE is thus currently used clinically for patients with a high risk of recurrence and high NLR. Under these circumstances, PA-TACE plays a largely positive role, as supported by the current research. However, in the absence of clinical consensus, the use of PA-TACE in patients with low NLR levels is controversial, and the present results suggested that PA-TACE may not benefit patients with a low NLR. This effect may occur because the cell-mediated immune response can still guarantee an anti-tumor effect under low-NLR conditions, while PA-TACE creates a suitable microenvironment for tumors, which instead leads to a poor prognosis. NLR may thus be used as an indicator to exclude patients who cannot benefit from PA-TACE.

After clarifying that patients with a lower NLR may not benefit from PA-TACE, we examined the relationships between several recognized high-risk recurrence factors of liver cancer and the NLR level by logistic regression. We found that a maximum tumor diameter >5 cm was the only factor correlated with a high NLR. However, the current study did not clarify the cause and effect relationship between these, and we therefore recommend PA-TACE treatment for patients with large liver tumors after surgery. Their highly malignant biological behavior means that it is very difficult to achieve radical resection of very large HCCs in an absolute sense, and residual tumor cells after surgery are almost inevitable. However, imaging and serological examinations showed that PA-TACE could destroy the remaining tumor cells and prevent or at least delay intrahepatic recurrence (29). The current study did not provide evidence to indicate that a lower NLR represented a low risk of recurrence, and we therefore cannot reach a conclusion on the benefit of PA-TACE in patients with a low risk of recurrence.

Our study had some limitations. First, it was a retrospective study with a limited number of cases in the subgroups. In addition, the relatively long follow-up interval was not conducive to accurate evaluation of RFS. We therefore anticipate the results of future multicenter, large-sample studies. Second, the differences in age and proportions of patients with liver cirrhosis between the subgroups when dividing patients treated with surgery and surgery plus PA-TACE in the high/low-NLR groups may have had an impact on our results. The proportions of patients over 55 years old and patients with liver cirrhosis were higher in the surgery-alone compared with the surgery plus PA-TACE subgroup. This may have been mainly because of considerations of patient safety, combined with clinical research evidence and treatment experience (41), given that PA-TACE is used cautiously in patients with severe preoperative cirrhosis, ascites before or after surgery, and poor postoperative physical condition. However, even if this tendency was similar in both pairs of subgroups, it may weaken our conclusions. Third, we did not summarize the most appropriate definitions of high NLR and low NLRs. There is currently no conclusive classification of the NLR, and the clinical definition and judgment of the NLR still relies on the experience of the doctors. We hope that future research can make further breakthroughs in this area.

## Conclusion

This study analyzed the relationship between preoperative NLR levels and the efficacy of PA-TACE in HCC patients, and confirmed that patients with a lower NLR before surgery were unlikely to benefit from PA-TACE, while PA-TACE could improve the prognosis of patients with a higher NLR. We therefore do not recommend PA-TACE in patients with HCC with low preoperative NLR values.

## Data Availability

The raw data supporting the conclusions of this article will be made available by the authors, without undue reservation.
